# A GO intervention program for enhancing elementary school children's cognitive functions and control abilities of emotion and behavior: study protocol for a randomized controlled trial

**DOI:** 10.1186/1745-6215-13-8

**Published:** 2012-01-12

**Authors:** Yoshiyuki Tachibana, Jiro Yoshida, Masahito Ichinomiya, Rui Nouchi, Carlos Miyauchi, Hikaru Takeuchi, Naoki Tomita, Hiroyuki Arai, Ryuta Kawashima

**Affiliations:** 1 Department of Child and Adolescent Psychiatry, University of Manchester and Manchester Academic Health Sciences Centre, Room 4.321, Psychiatry Research Group, 4th Floor (East), Jean McFarlane Building, University Place, University of Manchester and Manchester Academic Health Sciences Centre, Oxford Road, Manchester, M13 9PL, UK; 2 Department of Applied Brain Science, Smart Aging International Research Center, IDAC, Tohoku University, Japan, Seiryou-machi 4-1, Aoba-ku, Sendai-shi, Miyagi-ken 980-8575, Japan; 3 The Nihon Ki-in 9F, 1-7-20 Yaesu, Chu-ou-ku, Tokyo-to 103-0028, Japan; 4 Department of Functional Brain Imaging, IDAC, Tohoku University, Seiryou-machi 4-1, Aoba-ku, Sendai-shi, Miyagi-ken 980-8575, Japan; 5 Department of Geriatrics and Gerontology, IDAC, Tohoku University, Seiryou-machi 1-1, Aoba-ku, Sendai-shi, Miyagi-ken 980-8574, Japan

**Keywords:** Executive function, GO intervention program, Randomized controlled trial, Elementary school children, Development

## Abstract

**Background:**

Executive function is critical for children's healthy development. We propose an intervention program to enhance children's executive function using the game, GO. Many neuroimaging studies have revealed that playing GO is related to executive function. In addition, previous studies also revealed that executive function can be enhanced by training. We will perform a randomized controlled trial to investigate the effectiveness of a GO intervention group and a control group without intervention.

**Methods/Design:**

35 elementary school children aged 8 to 10 were recruited from Edogawa elementary school in Tokyo, Japan. They will be randomized into two groups; either the 5-week GO intervention group or no-intervention control group. We will ask the participants of the intervention group to join the GO course which will be held once every week for five weeks (total: six times). In the GO course, the children will be taught GO by the GO masters of the Nihon Ki-in and enjoy it for an hour. Besides the course, the participants will perform GO problems about twenty minutes a day, three times a week during the intervention period. We will use the Stroop task, the digit span, the Raven's colored progressive matrices, the Span-board task, and the Behavioral inhibition/behavioral activation scale for the outcome measures. Outcomes will be measured at a baseline (Assessment 1) and 5 weeks after the intervention program started (Assessment 2). The intervention group will be compared with the control group using one-way analyses of covariance with the difference between Assessment 1 and Assessment 2 measures as dependent variables and pretest scores as covariates.

**Discussion:**

To our knowledge, this study will be the first RCT to investigate the efficacy of a GO intervention program for elementary school children. If this intervention is effective, we will be able to take the next steps in making an educational program to enhance children's executive function and other cognitive abilities using GO. In addition, we further will investigate the transfer effects of the GO intervention program through executive function. We also will investigate neuroplasticity with the GO intervention using neuroimaging.

**Trial Registration:**

UMIN Clinical Trials Registry UMIN000006324

## Background

The abilities to control impulses make plans, and stay focused are not inborn. However, we are born with the potential to develop these abilities. Whether we can develop them or not depends on our experiences during infancy, throughout childhood, and into adolescence. A brain mechanism called executive function (EF) is deeply related to these skills [1]. It enables us to focus on multiple streams of information at the same time, organize and prioritize information, plan, monitor our progress, shift flexibly, and reflect on our activities [2]. The base of EF is acquired in early childhood [3], as the relevant circuits emerge, mature, and forge cortical interconnections [4]. These circuits are then refined and made more efficient during adolescence and into the early adult years [5]. It is also important to note that the brain regions and circuits associated with EF have extensive interconnections with the deeper brain structures that control the developing child's responses to threat and stress [6,7]. This implies that the developing EF system both influences and is affected by the young child's emotional and behavior control. Thus, the opportunity to further the EF's capacities in middle childhood and adolescence is critical to healthy cognitive and emotional development.

EF continues to mature from early childhood to mid-adolescence [8-12], reflecting a window of prolonged plasticity in underlying neural system [9]. Cognitive maturation in this system is believed to be subserved by changes in brain structure, including synaptic pruning [13] and myelination [14], that continue through childhood and begin to reach adult levels in mid-adolescence [5]. Luna et al. suggested that functional integration of prefrontal circuitry with a distributed circuitry may be compromised in autism undermining executive function based on their fMRI study [15]. They also suggested that the period of childhood to adolescence may be a window of opportunity to affect the course of development in a similar fashion as typically developing individuals and that some compensatory developmental progress may be possible [15].

The healthy development of EF skills can be supported with specialized practice and training [16]. Klingberg and his colleagues indicated that working memory training using a special computer-based program enhanced working memory (one of core factors of EF [17]), response inhibition, and reasoning in children with ADHD [18]. They applied the program to kindergarten children and their cognitive abilities showed enhancement [19,20]. However, as far as we know, there is no study about cognitive training for healthy children using a common activity easily available in daily life.

In this study, we propose an intervention program using GO, the concept of which is derived from knowledge of brain science and developmental psychology. GO is a traditional board game for two players that originated in China more than 2000 years ago. The game is noted for being rich in strategy despite its relatively simple rules. The game is played by two players who alternately place black and white stones on the vacant intersections (called "points") of a grid of 19 × 19 lines (beginners often play on smaller 9 × 9 and 13 × 13 boards). Stones act as markers, representing one's occupation of a particular point. The object of the game is to use one's stones to surround a larger portion of the board than one's opponent. The game has been enjoyed worldwide. An estimate done in 2003 places the number of GO players worldwide at approximately 42 million [21]. Since the black and white stones are identical except for their difference in color, the key factor in GO playing is spatial positioning [22]. Recent brain science studies revealed a deep relationship between playing GO and EF. Chen and his colleagues investigated brain activities while GO was being played using functional brain imaging (fMRI), and revealed enhanced activations in many cortical areas, such as the dorsal prefrontal, parietal, occipital, posterior temporal, primary somatosensory, and motor areas [22]. These regions are deeply related to EF. Park and his colleagues conducted voxel-based analyses of diffusion-tensor imaging data and found that, compared to inexperienced controls, long-term trained GO players developed larger regions of white matter with increased fractional anisotropy values in the frontal, cingulum, and striato-thalamic areas that are related EF [23]. We predict GO will be a highly effective EF training program for children.

### Aims and hypothesis

We aim to conduct the first randomized controlled trial of a GO intervention for elementary school children to assess the intervention's effectiveness in enhancing children's cognitive functions and control abilities of emotion and behavior. We hypothesize that the GO intervention program will enhance children's cognitive abilities, especially EF. We also hypothesize that it will affect children's control abilities of emotion and behavior. To test these hypotheses, we will conduct a randomized controlled trial. We predict that, compared to the controls, the children of the intervention group will gain better cognitive functions, especially EF and have better emotional and behavior control.

## Methods/Design

### Participants

Children in their second to fourth years of elementary school will be chosen as participants for two reasons. First, the Nihon Ki-in, which is the Japanese public GO association, holds GO courses for children in their second to fourth years of elementary school. Based on their experience in tutoring children in GO, they regard that span of ages as suitable for children to begin enjoying GO courses. The second reason for choosing the ages we have is because EF develops and become fractionated gradually from early childhood to adolescence [4]. It has been suggested that the systems for visuo-spatial working memory, a major component of EF [17], are fractionated to a greater extent in children aged 8 to 9 years [24]. GO is deeply related to visuo-spatial working memory [22]. Thus we regard children in their second to fourth year of elementary school to be profitable to our GO intervention program.

### Recruitment

The staff of the Nihon Ki-in will contact the president of Edogawa elementary school in Tokyo. The teachers at that school will then announce this study to their students and distribute a handout. Parents will receive an explanation of the study and a handout in a parent meeting. Then, the Nihon Ki-in staff will hold an explanatory meeting for the children and parent**s **regarding this study.

### Inclusion criteria

1) From age 8 to 10 years old (from the second to fourth grade of elementary school)

2) No experience playing GO

3) Interested in playing GO

4) Children able to attend all the classes of the GO courses (total of five times)

5) Children who have consent to do the GO tasks for about twenty minutes a day three times a week for five weeks at home

### Exclusion criteria

1) A current clinically severe illness or disorder making it impossible to perform the intervention program

### Study design

**Figure 1:**
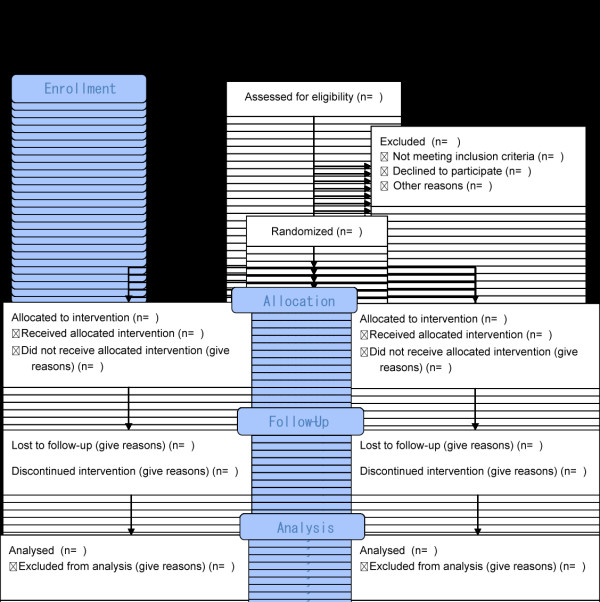
**Trial profile**.

This study is a randomized controlled trial (RCT) comparing our GO intervention program with unaltered, daily life in elementary school children aged 8 to 10 (See Figure 1). The project will run from September 2011 to November 2011. This period encompasses participant recruitment, baseline data collection, and intervention delivery. We will run the trial in accordance with the CONSORT statement [25]. Any selection bias will be reduced by rigorous application of a priori inclusion and exclusion criteria, and concealed allocation after consent is received. Though the participants cannot be made blind to the treatment allocation, the assessors will be kept blind. The assessors and data will be housed away from the sites where this intervention program will be performed. The randomization manager and the assessors will also remain in a separate area.

After consent is obtained, the Nihon Ki-in staff will allocate a sequential identification number and will provide a statistician at an independent site with each child's number. Stratified randomization will be used to ensure that the proportion of the grade is balanced between the two groups. This will be computer-generated. The statistician will then send the information of the allocation to the Nihon Ki-in staff. The assessors and supervising research staff will be unaware of this allocation. However, the allocation cannot be masked from the participants.

Strict separation will be kept between the assessment and the data; assessors will be located separately from the site where this trial will be performed. To avoid the effects of familiarity, the materials and location for assessment will be different from those for intervention. The rating of the questionnaire will be performed with an anonymised identification number assigned by an assessor from a different trial site, aware of neither the participant details nor the intervention status.

To measure the efficacy of our program, examinations for the children and questionnaires for the children and their mothers will be administered before (Assessment 1) and after (Assessment 2) the intervention group undergoes the intervention program. We will investigate the effectiveness of the program by comparing the results of Assessment 1 and Assessment 2 between the two groups. We will use the waiting-list control group design. After Assessment 2, the control group will participate in the intervention program. This will be done so that both groups can experience the program.

### Intervention

We will ask the participants of the intervention group to join the GO course which will be held once every week for five weeks (total of six times). In the GO course, the children will be taught GO by the GO masters of the Nihon Ki-in and enjoy playing it for an hour, using a 6 × 6 or a 9 × 9 board, both of which are suitable for GO beginners. In addition to this course, the participants will perform GO problems for approximately twenty minutes a day, three times a week during the intervention period. To make the problems suitable for the children's beginning-level, the GO problems will be chosen by the staff of the Nihon-Ki-in. The staff is well-experienced at designing beginner GO courses for tutoring elementary school children.

### Assessment tests

Data are collected using self- and parent-report questionnaires, as well as objective measures including face-to-face assessments and a parents' report questionnaire (all measures are outlined in Table 1). The outcomes will be measured at baseline 5 weeks after the intervention program starts. The measures are the following:

**Table 1:** Study measures and time-points

Measures	Answer	Time point	
		Baseline	5 weeks
Stoop task	Child	■	■
Digit span of the Wechsler Intelligence Scale for Children-III	Child	■	■
Raven's Coloured Progressive Matrix	Child	■	■
Span-board task of the Wechsler Adult Intelligence Scale-Revised	Child	■	■
The Behavioral Inhibition/Behavioral Activation Scales	Parent	■	■

1) Stroop task

This test is considered to be a measure selective attention, cognitive flexibility and processing speed, and it is used as a tool in the evaluation of EF [26,27]. We will use the New Stroop Test II (Japanese version) in this study [28], which is a matching type Stroop task requiring subjects to check whether their chosen answers are correct, unlike the traditional oral naming Stroop task. The test consists of two control tasks; a Stoop task, and a reverse Stroop task. The number of correct answer, reverse-Stroop interference rate and Stroop interference rate will be calculated.

2) Digit span in Wechsler Intelligence Scale for Children III

Digit span is a subtest of the Wechsler Intelligence Scale for Children III (WISC-III)[29], which is an individually administered intelligence test for children between the ages of 6 and 16. The test can be completed without reading or writing. In the digit span test, the subjects are orally given sequences of numbers and asked to repeat them, either as heard (forward) or in reverse order (backward). Research based on working memory theory [30,31] revealed that the forward digit span reflects the capabilities of phonological loop, and the backward digit span reflects EF [32,33].

3) Raven's Colored Progressive Matrices

This is a non-verbal multiple choice test. It measures the reasoning component of Spearman's *g *[34], which is often referred to as general intelligence [35]. The test was originally developed by John C. Raven in 1936 [36]. In each test item, the subject is asked to identify the missing element that completes a pattern. Most items are presented on a colored background to make the test visually stimulating for participants.

4) Span-board task

Span-board task are used from the Wechsler Adult Intelligence Scale-Revised (WAIS-RNI), as a Neuropsychological Instrument testing battery [37]. We will use this task to measure visuospatial working memory. Ten blocks are arranged in an irregular pattern in front of the subject. The testers point to a sequence of blocks and the subject then point to the same blocks in the same order (forward span-board task) or in the reverse version (backward span-board task).

5) The Behavioral Inhibition/Behavioral Activation Scales (Middle childhood version)

This scale was developed by Craver and colleagues [38] based on Gray's biopsychological theory of personality [39,40]. He hypothesized that personality has two systems which control activities, the Behavioral Inhibition System (BIS) and the Behavioral Activation System (BAS). This scale was standardized in Japanese [41]. The BIS is thought to be related to sensitivity to punishment as well as avoidance motivation while the BAS is thought to be related to sensitivity to reward as well as approach motivation. Additionally, the BAS system is thought to be related to dopaminergic pathways in the cortical-striatal-thalamic-cortical loop system associated with the orbitofrontal cortex (Depue & Collins, 1999). The BIS system, in contrast, is thought to reflect serotonergic functioning in the amygdale and septohippocampal system (Gray, 1987). EF plays an important role for BIS/BAS; individuals characterized by higher executive functions can regulate reactions to both BIS and BAS stimuli [42], and have better emotional and cognitive control [43]. We will assess the effectiveness of GO for BIS/BAS system using this scale.

### Outcomes

The primary outcome will come from the number of correct answer in the Stroop task. The secondary outcomes will come from reverse-Stroop interference rate and Stroop interference rate in the Stroop task; the scores of the forward and backward of the digit span test in the WISC-III; the total score of the Raven's Colored Progressive Matrix; the scores of the forward and backward of the Span-board task of the WAIS-RNI; and the subscales of the Behavioral Inhibition/Behavioral Activation Scales: BIS, BAS Reward Responsiveness, BAS Drive, BAS Fun Seeking.

### Fidelity assessment

We will perform the fidelity assessment of this program for the intervention group with a self-answered questionnaire in Assessment 2. We will ask participants, "How often did you play GO at home?". The participants will select one among the following alternatives, "1. Almost every day, 2. 4 or 5 times a week, 3. 3 times a week, 4. 2 or 3 times a week, 5. 2 times a week, 6. 1 times a week, 7. Less than 1 time a week".

### Statistical analysis

To assess whether there are any significant differences in the Assessment 1 - Assessment 2 changes between the intervention group and the control group, the intervention group will be compared with the control group using one-way analyses of covariance (ANCOVAs) with the difference between Assessment 1 and Assessment 2 measures as dependent variables and pretest scores as covariates to exclude the possibility that any pre-existing difference in the measures between the groups affected the results of each measure.

### Sample size

In order to detect a 0.55 effect size shift of the number of correct answer in the Stroop task between the intervention and control group with 80% power and at a significance level of 0.05 for the post-pre changes, we will require 14 children in each group (total 29 children). Assuming a 17.1% dropout rate (3 children for each group), this study needs a final sample size of 35 children. This sample size will also enable us to detect shifts in other secondary outcomes between the two groups.

## Discussion

GO will be easy to use as a cognitive intervention tool. To our knowledge, no intervention research for enhancing children's cognitive abilities using common tools in daily life has been performed. This study is the first RCT to investigate the efficacy of a GO intervention program for elementary school children. We will investigate whether our GO intervention program enhances the children's cognitive abilities and mental health. If this intervention is effective, we will be able to take the next steps in making an educational program to enhance children's EF and other cognitive abilities using GO. In addition, we will further investigate the transfer effects of the GO intervention program through EF. We also will investigate neuroplasticity resulting from the GO intervention using neuroimaging.

## Trial Status

The trial is ongoing.

## List of abbreviations

ANCOVAs: one-way analyses of covariances; EF: executive function; RCT: randomized controlled trial; WISC-III: Wechsler Intelligence Scale for Children III; WAIS-RNI: Wechsler Adult Intelligence Scale-Revised; BIS: Behavioral Inhibition System; BAS: Behavioral Activation System.

## Competing interests

The authors declare that they have no competing interests.

## Authors' contributions

YT, JY, RN, and RK contributed to the overall design and conception of the study. YT drafted this manuscript. MI contributed to the GO course. JY contributed to developing the Go course program, the GO problems, and the manual of the program. NT and HA will also contribute to the randomization procedure. HT and CM provided advice regarding measurement and assessment. All authors read and approved the final manuscript.
